# From serum inflammatory markers to fluid, tissue, and molecular assays: current advances in the laboratory diagnosis of bone and joint infections

**DOI:** 10.3389/fcimb.2026.1865643

**Published:** 2026-07-14

**Authors:** Jinglin Li, Shilan Lian, Yiming Liu, Xuxu Yang, Datao Liu, Ji Chen, Huazhang Xiong

**Affiliations:** 1 Department of Orthopedics, Affiliated Hospital of Zunyi Medical University, Zunyi, China; 2 Department of Clinical Laboratory, People’s Hospital of Honghuagang District, Zunyi, China; 3 Department of orthopedics, Guangxi Medical University Affiliated Liuzhou People’s Hospital, Liuzhou, China; 4 Joint Orthopaedic Research Center of Zunyi Medical University, University of Rochester Medical Center, Zunyi, China

**Keywords:** bone and joint infections, fracture-related infection, laboratory diagnosis, molecular diagnostics, periprosthetic joint infection, synovial biomarkers

## Abstract

Bone and joint infections (BJIs), including periprosthetic joint infection (PJI), fracture-related infection (FRI), and osteomyelitis, present persistent diagnostic challenges driven by biofilm formation and a high incidence of culture-negative cases. Traditional diagnostic modalities relying on peripheral serum markers and conventional cultures are often limited by insufficient specificity or prolonged turnaround times. This narrative review critically evaluates recent advances in laboratory diagnosis for bone and joint infections, with particular attention to disease-specific applicability across periprosthetic joint infection, fracture-related infection, native vertebral osteomyelitis, diabetic foot osteomyelitis, and other osteomyelitis-related conditions. Current evidence indicates that while traditional serum inflammatory markers are valuable for initial screening, their susceptibility to aseptic inflammatory confounders precludes standalone diagnostic confirmation. In contrast, localized sampling demonstrates significant superiority: novel synovial fluid biomarkers, notably calprotectin and alpha-defensin, accurately reflect the infection microenvironment and offer exceptional diagnostic specificity. At the tissue level, the integration of multiple deep-tissue sampling with preprocessing techniques like sonication has substantially enhanced the recovery of occult biofilm-encased pathogens. Furthermore, targeted and untargeted molecular assays, including multiplex PCR panels, broad-range bacterial PCR, amplicon-based sequencing, and untargeted shotgun metagenomic sequencing, have expanded the diagnostic toolkit for culture-negative, low-virulence, and polymicrobial infections. The diagnostic framework for BJIs has decisively shifted from the pursuit of a solitary “silver bullet” marker toward multimodal, culture-independent assay panels and artificial intelligence-assisted risk stratification algorithms. Future clinical breakthroughs will depend heavily on the global standardization of disease definitions, robust external validation of predictive models, and the seamless integration of advanced laboratory techniques into multidisciplinary team (MDT) workflows.

## 1 Introduction

Bone and joint infections (BJIs) are complex infectious diseases involving bone tissue, the joint cavity, and implant interfaces. They encompass a broad spectrum of conditions, including periprosthetic joint infection (PJI), fracture-related infection (FRI), pyogenic osteomyelitis, and native vertebral osteomyelitis (NVO), and represent some of the most destructive and difficult-to-manage complications in modern orthopedic surgery and trauma care (Isler et al., 2025; Matsuo et al., 2025). With the acceleration of global population aging, the substantial increase in primary joint arthroplasty procedures, and the growing number of interventions following high-energy trauma, the incidence of BJIs has shown an upward trend, posing major challenges to infection control, long-term functional recovery, and healthcare expenditure (Foster et al., 2024). Beyond imposing a heavy economic burden on healthcare systems, BJIs can also have profound adverse effects on patient prognosis. Some cohort studies have reported that the five-year survival of patients with PJI may be even lower than that of patients with several common malignancies, such as breast cancer, prostate cancer, or malignant melanoma. Accordingly, BJIs are increasingly recognized as a major public health and patient-safety concern, and their associated mortality risk makes them among the most feared complications after total joint arthroplasty (Jensen et al., 2025).

Traditionally, the laboratory diagnosis of BJIs has relied heavily on microbial culture and empirically used serum inflammatory markers (Osmon et al., 2013; McNally et al., 2021). Although different disease subtypes vary in anatomical location, host background, and surgical context, they share a common pathological feature: pathogens can form biofilms on bone surfaces, necrotic tissue, or implant materials, thereby inducing a low-grade, occult, and persistent infectious state that markedly compromises the diagnostic performance of conventional culture and traditional inflammatory markers (Masters et al., 2022). Within the biofilm microenvironment, some pathogens may enter a viable but non-culturable (VBNC) state through transcriptional and metabolic reprogramming. Under conditions of nutrient limitation, hypoxia, and antimicrobial pressure, their metabolic activity decreases and proliferation becomes arrested, reducing the detection rate of conventional culture methods (Pasquaroli et al., 2013; Li et al., 2014). VBNC bacteria are considered one potential contributor to culture-negative orthopedic infections, although culture negativity is usually the result of multiple interacting factors. Current evidence indicates that culture-negative cases account for approximately 5%–42% of PJI and 10%–22% of FRI cases (Kalbian et al., 2020; Wong et al., 2022; Seidelman and DeBaun, 2025). Such diagnostic delays and false-negative pathogen detection may postpone the optimal timing of targeted antimicrobial therapy and contribute to the failure of early surgical strategies, including debridement, antibiotics, and implant retention (DAIR) (Maritati et al., 2025).

**Figure 1:**
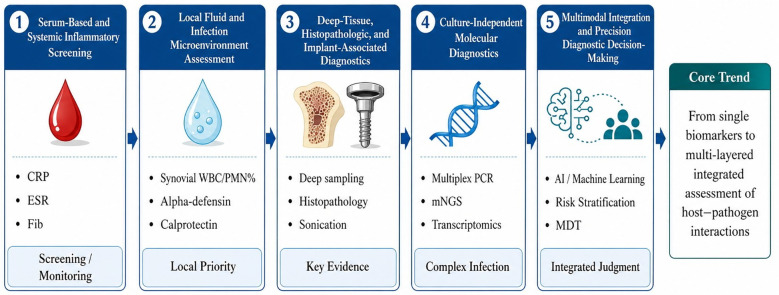
Disease- and specimen-stratified framework for laboratory diagnosis of bone and joint infections.

To address this long-standing obstacle in clinical decision-making, recent high-quality systematic reviews and the 2025 International Consensus Meeting on Musculoskeletal Infection (ICM 2025) have collectively highlighted an ongoing shift in the laboratory diagnostic paradigm for BJIs (Al-Jabri et al., 2024; Martinazzi et al., 2025). The current diagnostic framework is moving beyond a single-dimensional strategy of “pathogen hunting” toward a more comprehensive assessment of host–pathogen interactions (Maritati et al., 2025). This transition spans the redefinition of disease-specific thresholds for peripheral serum biomarkers, the simultaneous detection of host defense peptides and pathogen-derived metabolites within the synovial microenvironment, and the increasing application of targeted multiplex polymerase chain reaction (multiplex PCR), metagenomic next-generation sequencing (mNGS), and host transcriptomic profiling. Together, these advances are improving both the timeliness and precision of laboratory diagnosis (Olearo et al., 2025). In addition, artificial intelligence and machine learning are being increasingly explored to integrate serum, synovial fluid, histopathological, and molecular data, thereby improving risk stratification and diagnostic consistency in complex cases. Accordingly, the laboratory diagnosis of BJIs is gradually shifting from a traditional model based on isolated culture results or single biomarkers toward a refined diagnostic pathway centered on local sampling, molecular identification, and multimodal integration (Li et al., 2026). This review is not intended to treat all BJIs as a single homogeneous diagnostic entity. Instead, we use PJI as the best-studied reference condition for many laboratory assays, while separately discussing FRI, NVO, diabetic foot osteomyelitis, and other osteomyelitis-related conditions when disease-specific evidence is available. These entities differ substantially in anatomical location, biofilm burden, specimen accessibility, microbiological spectrum, reference standards, and clinical consequences of delayed diagnosis. Therefore, diagnostic thresholds and test performance derived from one disease entity should not be automatically extrapolated to another. In the following sections, we distinguish established clinical applications from emerging or exploratory uses and highlight where evidence remains limited, non-transferable, or requires disease-specific validation. (**Figure 1**).

## 2 Systematic reappraisal and specificity assessment of serological biomarkers

### 2.1 Clinical repositioning and dynamic value of conventional acute-phase proteins: CRP and ESR

C-reactive protein (CRP) and erythrocyte sedimentation rate (ESR) are among the most established and widely used serological screening markers in clinical diagnostic pathways. These markers usually show nonspecific elevations in response to injury, surgery, or other acute inflammatory conditions. Conversely, their levels may remain within the normal range in chronic or late-onset infections (Chen et al., 2021; Lourtet-Hascoët et al., 2025). In large cohort studies evaluating PJI and FRI, CRP and ESR have demonstrated high negative predictive value (NPV), helping clinicians exclude infection during the initial screening stage. A receiver operating characteristic (ROC) curve analysis in patients with PJI showed that serum CRP remained one of the best-performing conventional serum markers, with an area under the curve (AUC) greater than 0.90 and an optimal sensitivity of up to 0.92 (Sun et al., 2025). A retrospective study of PJI further evaluated the diagnostic accuracy of different biomarker combinations in chronic PJI and found that combining ESR, CRP, the percentage of polymorphonuclear neutrophils (PMN%), and the platelet volume ratio (PVR) improved the diagnostic accuracy of chronic PJI in patients undergoing total hip or knee arthroplasty revision (Denyer et al., 2023). However, a systematic review and meta-analysis focusing on late and chronic PJI indicated that although serum CRP, fibrinogen, and interleukin-6 (IL-6) showed the best overall diagnostic performance, their absolute accuracy remained insufficient for independently confirming or completely excluding PJI because of substantial inter-study heterogeneity and biases introduced by different infection definitions (Sigmund et al., 2025c). Therefore, international consensus recommendations emphasize that serum inflammatory markers should be interpreted only as auxiliary signals within a multidimensional diagnostic framework. In patients with a high clinical suspicion of infection, more invasive joint aspiration and synovial fluid analysis should still be performed even when CRP, IL-6, and ESR are all within normal ranges. In diabetic foot osteomyelitis (DFO), a distinct infectious condition characterized by the coexistence of peripheral vascular insufficiency and chronic tissue ulceration, the clinical threshold of ESR is being redefined. A 2025 meta-analysis using hierarchical summary receiver operating characteristic (HSROC) modeling and generalized linear mixed models (GLMMs) evaluated studies including 1,674 patients with DFO and identified 51.6 mm/h as the optimal overall ESR threshold for DFO screening, with a sensitivity of 0.80 and a specificity of 0.67. Compared with the high-specificity threshold of 70 mm/h widely recommended in previous international guidelines, which showed a sensitivity of only 0.61 and a specificity of 0.83, the 51.6 mm/h threshold may substantially reduce missed diagnoses at the cost of a modest reduction in specificity (Liu et al., 2025). In the clinical management of DFO, missed diagnosis may delay timely deep debridement and increase the risk of irreversible amputation; therefore, a more sensitive threshold may have greater clinical and public health relevance. In addition, in patients with DFO, CRP, ESR, and procalcitonin (PCT) have limited cross-sectional value for distinguishing isolated soft-tissue infection from osteomyelitis at initial presentation. However, their serial dynamic monitoring remains valuable for evaluating antibiotic response, predicting ulcer-healing trajectories, and informing decisions regarding treatment discontinuation (Gureh et al., 2025). Because the diagnostic performance of systemic biomarkers varies substantially by infection entity, anatomical site, infection chronicity, specimen definition, and reference standard, the values summarized in **Table 1** are reported only within the disease context of the cited studies. They should not be interpreted as universal estimates for all BJIs. This distinction is particularly important for markers such as CRP, ESR, fibrinogen, D-dimer, and NLR, whose apparent accuracy may differ between PJI, FRI, DFO, NVO, and non-implant-associated osteomyelitis.

**Table 1:** Disease-specific interpretation of selected serological biomarkers in bone and joint infections.

Biomarker	Sample type	Disease entity of the cited evidence	Evidence summary/reported evidence context	Main clinical interpretation	Key limitations and transferability
CRP	Serum	PJI	Selected PJI studies report favorable screening performance, including high AUC values in some cohorts; however, systematic reviews emphasize substantial heterogeneity and insufficient standalone diagnostic accuracy	Useful first-line screening and rule-out adjunct in suspected PJI	Nonspecific elevation after surgery, trauma, inflammatory arthritis, malignancy, or other aseptic inflammatory conditions. Normal CRP does not exclude chronic or low-grade infection
ESR	Serum	PJI/FRI	Widely included in composite diagnostic frameworks, but no universal threshold applies across PJI and FRI	Conventional screening marker, especially when interpreted together with CRP and local findings	Slow dynamic response and poor specificity. Should not be used as an independent confirmatory test
ESR	Serum	DFO	A DFO-focused meta-analysis reported an optimal ESR threshold of approximately 51.6 mm/h, with sensitivity of 0.80 and specificity of 0.67; the traditional 70 mm/h threshold showed lower sensitivity but higher specificity	Useful screening adjunct for DFO, where avoiding missed diagnosis may be clinically important	DFO-specific threshold. It should not be transferred directly to PJI, FRI, NVO, or other osteomyelitis entities
Fibrinogen	Plasma/serum	PJI	PJI-focused cohort studies and diagnostic models suggest diagnostic value comparable to CRP and ESR, with high AUC values reported in selected cohorts	Adjunctive preoperative marker for suspected PJI	Systemic coagulation/inflammatory marker. Performance varies by infection stage, reference standard, and comorbid thromboinflammatory conditions
D-dimer	Plasma/serum	PJI	A 2025 PJI meta-analysis reported moderate pooled diagnostic performance, with sensitivity of 0.74, specificity of 0.72, and AUC of 0.79; heterogeneity was influenced by sample type, infection stage, and reference standard	Potential adjunctive triage or rule-out marker in selected PJI settings	Affected by thrombosis, trauma, surgery, malignancy, and systemic inflammation. Not suitable as a standalone diagnostic marker
D-dimer	Plasma/serum	Primary osteomyelitis/non-implant-related trauma	In non-PJI cohorts, abnormal D-dimer elevation has not shown a consistent association with positive bacterial culture or implant-associated osteomyelitis	Limited value outside selected PJI contexts	Highlights the risk of extrapolating PJI-derived evidence to broader osteomyelitis populations
NLR	Peripheral blood	FRI	One FRI-focused study reported that NLR >2.45 was associated with infectious biopsy findings, but this threshold requires external validation	Low-cost adjunctive screening or risk-stratification marker for FRI	Influenced by age, trauma, systemic inflammation, immune status, and perioperative stress. Not a universal diagnostic cut-off
NLR	Peripheral blood	Pediatric MSKI/perioperative orthopedic cohorts	Pediatric and perioperative studies suggest that NLR may reflect systemic immune stress and disease severity, but no universal diagnostic threshold has been established	Adjunctive severity or risk marker rather than a confirmatory diagnostic test	Age-related immune variation and systemic inflammatory conditions substantially affect interpretation

The diagnostic performance values summarized in this table are disease- and study-context-specific estimates rather than universal parameters for all bone and joint infections. Sensitivity, specificity, AUC, and optimal thresholds may vary according to disease entity, specimen type, joint site, infection chronicity, organism virulence, prior antimicrobial exposure, assay platform, and diagnostic reference standard. Values are retained only when supported by cited meta-analyses or clearly defined disease-specific studies; otherwise, qualitative evidence summaries and clinical positioning are provided.

### 2.2 Coagulation-derived biomarkers: diagnostic performance of D-dimer and fibrinogen

Systemic inflammatory responses are often accompanied by activation of the coagulation cascade and dysregulation of the fibrinolytic system. As direct products or mediators of this pathophysiological process, serum D-dimer and fibrinogen have increasingly been incorporated into the diagnostic evaluation of orthopedic infections in recent years. Fibrinogen has shown relatively reliable diagnostic performance in PJI. A multicenter retrospective study reported that plasma fibrinogen had favorable diagnostic value for PJI, with good sensitivity and specificity comparable to those of conventional markers, including CRP and ESR (Li et al., 2019). Multiple retrospective cohort studies and diagnostic models have further confirmed that, before revision arthroplasty, serum fibrinogen can achieve a diagnostic AUC of up to 0.917. Its sensitivity and specificity for identifying culture-positive infection are highly comparable to those of CRP and may even be superior in certain subgroups, supporting its use as a valuable adjunctive marker in routine preoperative assessment for suspected PJI (Chen et al., 2021). By contrast, the clinical utility of D-dimer remains controversial. Since its inclusion as a minor diagnostic criterion for PJI by the International Consensus Meeting (ICM) in 2018, numerous studies have attempted to validate its diagnostic value. A retrospective analysis evaluating serum D-dimer as a diagnostic indicator for PJI reported relatively high sensitivity and specificity, reaching 87.50% and 89.19%, respectively (Hu et al., 2020). However, a recent 2025 meta-analysis including 30 studies and 6,444 patients showed that the pooled sensitivity and specificity of plasma or serum D-dimer for diagnosing PJI were 0.74 and 0.72, respectively, with an AUC of 0.79. Although the certainty of evidence was rated as moderate according to the GRADE framework, multivariable regression analysis indicated that sample type, infection stage, and heterogeneity in reference standards substantially affected its diagnostic stability (Luo et al., 2026). In addition, a prospective study of 502 patients undergoing revision hip or knee arthroplasty found that plasma D-dimer was non-inferior to serum CRP and ESR in diagnosing PJI and may serve as a useful adjunctive screening marker in patients undergoing revision total joint arthroplasty (Tarabichi et al., 2023). However, in general cohorts involving acute or chronic primary osteomyelitis and non-implant-related trauma, abnormal elevation of D-dimer (>0.5 mg/L) was not significantly associated with positive bacterial culture or implant-associated osteomyelitis (Ali et al., 2024). Based on the current low- to moderate-certainty evidence, D-dimer is best positioned as an initial triage marker or adjunctive rule-out test for PJI, particularly in low-prevalence settings.

### 2.3 Host systemic immune-inflammatory index: predictive application of NLR

Derived indices based on complete blood counts, particularly the neutrophil-to-lymphocyte ratio (NLR), have been shown to be inexpensive and robust biological parameters reflecting systemic immune stress and inflammatory burden in the host (Salimi et al., 2024; Wang et al., 2025). NLR has been identified as an independent risk factor for post-traumatic infection, with greater predictive value in patients older than 60 years. In orthopedic surgery, its application has mainly been investigated as a prognostic marker for joint infection (Nairn et al., 2024). A retrospective study further demonstrated that elevated preoperative NLR was independently associated with increased mortality after proximal femoral fracture surgery, especially in very elderly patients. Given its simplicity and widespread availability, NLR may serve as a useful adjunctive tool for early perioperative risk stratification (Civinini et al., 2026). A recent non-invasive diagnostic study focusing on fracture-related infection (FRI) suggested that NLR imbalance, driven by neutrophil chemotactic aggregation and lymphocyte depletion or apoptosis, may represent an important systemic reflection of deep bone infection. By comparing preoperative inflammatory indices with deep tissue biopsy findings in a large cohort, the study identified an optimal diagnostic threshold of NLR > 2.45. At this cut-off value, 92.6% of patients with infectious purulent biopsy findings showed elevated preoperative NLR, whereas this proportion was only 7.7% among patients with aseptic biopsy findings, with a reported odds ratio of 150 (p < 0.001). These findings support the potential value of NLR as a non-invasive early screening marker for FRI, although its diagnostic role still requires validation in broader clinical settings (Lapica et al., 2026). In addition, a meta-analysis involving 10,015 patients undergoing hip fracture surgery found that significantly elevated preoperative NLR, with reported thresholds ranging from 3.2 to 8.4, was not only associated with the prediction of occult infection but also served as an independent prognostic risk factor for mid- to long-term all-cause mortality after surgery, particularly beyond one year (Liu et al., 2025). In pediatric musculoskeletal infection (MSKI), accurate assessment of disease presence and severity is essential for effective triage and treatment. A retrospective cohort study based on a pediatric orthopedic consultation database for suspected MSKI from January 2013 to July 2022 found that white blood cell count had limited predictive value for the presence and severity of MSKI because of substantial age-related variability. In contrast, NLR showed lower age-related variability and may therefore represent a more reliable indicator for diagnosing MSKI and assessing disease severity in children (Hou et al., 2025). Overall, serum biomarkers should be interpreted differently across BJI subtypes. In PJI and FRI, CRP and ESR are most useful as initial screening or rule-out adjuncts but lack sufficient specificity for independent confirmation, particularly in patients with recent surgery, trauma, inflammatory arthritis, or other aseptic inflammatory conditions. In NVO, inflammatory markers are valuable for raising suspicion and monitoring response, but microbiological confirmation still relies primarily on blood cultures and image-guided biopsy when feasible. In diabetic foot osteomyelitis, ESR thresholds may have greater screening relevance than in implant-associated infections, but serum markers alone remain insufficient to distinguish soft-tissue infection from bone involvement. Thus, serum biomarkers are best viewed as disease-contextualized triage tools rather than universal diagnostic criteria for all BJIs. (**Table 1**).

## 3 Multidimensional profiling of synovial fluid and local body fluid assays: direct reflection of the infection microenvironment

### 3.1 Host defense peptides: diagnostic accuracy of alpha-defensin and calprotectin

Compared with peripheral serum markers, synovial fluid, bone marrow cavity effusion, and local aspirates from infectious lesions can more directly reflect pathological alterations within the target-organ microenvironment, although their interpretation may still be influenced by systemic inflammatory status and sampling conditions (Li et al., 2020). Synovial white blood cell (WBC) count and polymorphonuclear neutrophil percentage (PMN%) remain the most established and widely used cytological parameters in synovial fluid analysis for suspected PJI. Previous meta-analytic evidence supports their clinically useful diagnostic performance after total hip or knee arthroplasty; however, their interpretation is threshold-dependent and may be affected by joint site, infection chronicity, causative organism, prior antibiotic exposure, sample quality, and aseptic inflammatory mimics such as inflammatory arthritis, crystal-induced arthritis, and metallosis. Recent methodological reassessment further cautions that WBC count and PMN% should not be used as standalone rule-in tests, but should be interpreted within multimodal diagnostic frameworks integrating clinical findings, serum markers, microbiology, histopathology, and other synovial biomarkers. Within this broader framework, modern synovial fluid analysis has increasingly expanded to include local host-defense peptides, pathogen-specific metabolites, and physicochemical properties of synovial fluid (Qu et al., 2014; Ottink et al., 2019; Parthasarathi et al., 2026). Alpha-defensin (AD) is a potent antimicrobial peptide synthesized and released in large quantities by neutrophils during pathogen phagocytosis or intense stimulation by bacterial antigens. Its concentration can increase substantially in infected synovial fluid (Marson et al., 2018; Paul et al., 2025). A meta-analysis evaluating the diagnostic accuracy of methods used for pre-revision diagnosis of periprosthetic joint infection (PJI) assessed the diagnostic value of alpha-defensin by calculating sensitivity, specificity, diagnostic odds ratio, summary receiver operating characteristic curve area under the curve (AUC), positive likelihood ratio, and negative likelihood ratio, and found that alpha-defensin has considerable potential for diagnosing PJI (Xie et al., 2017). A 2025 systematic review including 51 studies and strictly following PRISMA guidelines showed that synovial AD testing is mainly performed using two approaches: laboratory-based enzyme-linked immunosorbent assay (AD-ELISA) and rapid point-of-care lateral flow immunoassay (AD-LF). The data indicated that AD-ELISA achieved excellent diagnostic performance, with a pooled sensitivity of 87.8% and a specificity of 97.9%. Although AD-LF has been widely adopted because of its convenience and retained high specificity of 97.0%, its sensitivity was lower, at 81.8% (Paul et al., 2025). In patients with complex inflammatory comorbidities such as rheumatoid arthritis or crystal-induced arthritis, conventional serological markers may be unreliable, whereas synovial AD and leukocyte count have shown stronger resistance to inflammatory interference and may provide more accurate information for differential diagnosis (Yilmaz et al., 2023). Calprotectin, another calcium-binding heterodimeric protein highly enriched in the cytoplasm of neutrophils, has intrinsic antimicrobial properties. It inhibits bacterial growth by binding essential metal ions and participates in inflammatory responses and leukocyte migration (Sejersen et al., 2025). It has rapidly emerged as a promising biomarker in the diagnosis of PJI and FRI. Integrated bulk RNA sequencing and single-cell RNA sequencing (scRNA-seq) analyses of the immune microenvironment in PJI synovial tissue have shown that calprotectin is significantly upregulated in PJI synovium and is mainly expressed by myeloid-derived suppressor cells (MDSCs) (Tuo et al., 2026). Several systematic reviews and meta-analyses have suggested that synovial calprotectin testing is an accurate method for diagnosing hip and knee PJI. Whether measured by enzyme-linked immunosorbent assay (ELISA) or lateral flow test (LFT), synovial calprotectin appears to perform better than conventional synovial WBC count in both ruling out and confirming chronic hip and knee PJI (Alkadhem et al., 2024; Festa et al., 2024). Comparative analyses of multiple synovial biomarkers have further shown that calprotectin has the highest overall accuracy, followed by alpha-defensin, leukocyte esterase, and synovial CRP, with reported sensitivity ranging from 78% to 92% and specificity from 90% to 95% (Vale et al., 2023). Because calprotectin is highly stable at room temperature and requires no complex sample pretreatment, it is particularly suitable for integration into lateral-flow point-of-care testing (POCT) kits. This rapid testing capability may substantially shorten the interval from aspiration to clinical decision-making, especially in emergency departments, outpatient clinics, and intraoperative settings where large biochemical platforms may not be readily available (Altsitzioglou et al., 2024). Disease-specific interpretation is essential for synovial host-defense peptide assays. Alpha-defensin and calprotectin currently have their strongest evidence base in PJI, particularly in hip and knee arthroplasty settings where synovial fluid can be reliably obtained and interpreted within established diagnostic frameworks. In FRI, evidence for these biomarkers is more limited and remains less standardized because available specimens may include peri-implant fluid, wound exudate, or intraoperative tissue rather than native synovial fluid. For NVO and many non-joint forms of osteomyelitis, synovial biomarkers are generally not directly applicable because the relevant diagnostic specimens are blood cultures, CT-guided biopsy material, vertebral tissue, or paraspinal collections. Therefore, alpha-defensin and calprotectin should be presented as clinically mature in PJI, potentially informative but still investigational in selected FRI contexts, and largely non-transferable to NVO unless future disease-specific sampling strategies and thresholds are established. In addition to host-response biomarkers, pathogen-directed antigen immunoassays represent another local fluid-based strategy for rapid pathogen-associated signal detection. These assays use antibodies to detect predefined microbial antigens in synovial fluid or other local specimens and may provide rapid, culture-independent information when integrated with conventional synovial fluid analysis. A nationwide study of microorganism antigen testing supported its potential value as a component of preoperative synovial fluid assessment. However, the diagnostic breadth of antigen immunoassays is inherently limited by panel design, and negative results cannot exclude infection caused by off-panel organisms. Recent evidence also suggests that commercial synovial antigen testing does not provide sufficient additional benefit to replace traditional culture. Therefore, pathogen antigen testing should be viewed as a complementary component of selected multi-biomarker diagnostic platforms rather than a standalone substitute for culture, molecular testing, or histopathology (Toler et al., 2023; Tarabichi et al., 2024). (**Table 2**).

**Table 2:** Advantages, indications, and limitations of synovial fluid and local body fluid assays in the diagnosis of bone and joint infections.

Assay	Sample type	Main applicable scenarios	Evidence summary/reported evidence context	Main advantages	Main limitations	Optimal clinical positioning
Synovial WBC/PMN%	Synovial fluid/local joint aspirate	First-line local inflammatory assessment in suspected PJI and septic arthritis	Well-established conventional synovial biomarkers. Recent reviews and corrected meta-analyses support their diagnostic value, but emphasize that thresholds and performance vary by joint site, infection chronicity, organism type, aspiration quality, and reference standard	Rapid, inexpensive, widely available, and directly reflects local neutrophil-dominant inflammatory burden	Susceptible to blood contamination, low-volume aspirates, crystal-induced arthritis, inflammatory arthritis, metallosis/adverse local tissue reaction, recent surgery or trauma, prior antibiotic exposure, and low-virulence chronic infection	Foundational local inflammatory layer; best used together with clinical assessment, serum markers, synovial biomarkers, culture, histopathology, and molecular assays rather than as a standalone rule-in test
Alpha-defensin (AD-ELISA)	Synovial fluid	PJI, especially diagnostically challenging cases or patients with concomitant inflammatory joint disease	A recent systematic review reported strong diagnostic performance for AD-ELISA, with pooled sensitivity of 87.8% and specificity of 97.9%; however, estimates remain dependent on study design, population, diagnostic criteria, and specimen quality	High overall diagnostic accuracy in PJI-focused studies and relatively good resistance to some inflammatory confounders	Higher cost, laboratory-based workflow, and possible false-positive or false-negative results in selected contexts such as metallosis/adverse local tissue reaction, inflammatory comorbidities, low-virulence infection, or poor-quality aspirates	High-value local adjunctive biomarker within composite PJI diagnostic frameworks rather than a standalone confirmatory test
Alpha-defensin (AD-LF)	Synovial fluid	Rapid point-of-care or intraoperative assessment of suspected PJI	The same systematic review reported high specificity for AD-LF, but lower sensitivity than AD-ELISA. In the cited analysis, specificity was 97.0% and sensitivity was 81.8%, although values vary across studies and reference standards	Convenient, rapid, and suitable for POCT or intraoperative decision support	Lower sensitivity than ELISA in several analyses; negative results should not independently exclude infection when clinical suspicion remains high	Rapid adjunctive test to support, but not replace, synovial WBC/PMN%, culture, histopathology, and composite diagnostic criteria
Calprotectin	Synovial fluid	Local diagnosis of PJI and selected FRI-related local fluid settings	Comparative analyses and meta-analyses report favorable diagnostic performance in hip and knee PJI, with reported sensitivity approximately 78%–92% and specificity approximately 90%–95%. Evidence in FRI remains less standardized	Stable at room temperature, requires simple sample preparation, suitable for POCT, and shows high overall accuracy in PJI-focused studies	Thresholds, assay platforms, and disease-specific applications still require standardization; evidence is strongest in PJI and less mature in FRI or non-joint osteomyelitis	Preferred local biomarker candidate for rapid PJI assessment, but still best interpreted within composite diagnostic frameworks
D-lactate	Synovial fluid	Rapid preoperative or intraoperative adjunctive diagnosis of PJI	A meta-analysis reported pooled sensitivity of 82%, specificity of 76%, diagnostic odds ratio of 14.18, and AUC of 0.84 for synovial D-lactate in PJI diagnosis	Directly reflects pathogen metabolism; requires small sample volume; short turnaround time and relatively low cost	Lower specificity than some host-defense peptide markers; may be influenced by pathogen spectrum, sample handling, and local biochemical conditions	Supplementary pathogen-metabolism biomarker, particularly useful when combined with host-response markers and conventional synovial analysis
Pathogen antigen immunoassays	Synovial/local fluid	Selected PJI-focused multi-biomarker platforms	Studies of microorganism antigen testing suggest potential value for rapid detection of predefined microbial antigenic targets, but recent evidence also indicates that commercial synovial antigen testing does not replace traditional culture	Rapid, culture-independent, and potentially useful when integrated with host-response biomarkers	Limited by antigen panel breadth; negative results cannot exclude off-panel organisms; clinical utility depends on platform design and target coverage	Complementary pathogen-directed signal within selected multi-biomarker platforms rather than a standalone substitute for culture, molecular testing, or histopathology
Combined pH+glucose+D-lactate	Synovial fluid	Rapid local biochemical assessment of suspected PJI, especially in resource-limited settings	A prospective cohort study reported that combining D-lactate with synovial pH and glucose reflected the infected biochemical microenvironment; when D-lactate was set at 8.45 mmol/L and combined with reduced pH, the model showed an NPV of approximately 84%	Low cost, biologically coherent, rapid, and potentially suitable for primary-care or resource-limited settings	Evidence level remains lower than that of established synovial biomarkers; affected by sample quality, handling conditions, and local validation	Rapid auxiliary biochemical combination, especially where advanced molecular or immunoassay platforms are not readily available
Synovial CRP/leukocyte esterase	Synovial fluid	Combined use with other local biomarkers in suspected PJI	Generally easy to integrate into synovial fluid workflows, but comparative analyses suggest lower overall accuracy than calprotectin and alpha-defensin	Simple, accessible, and compatible with existing diagnostic pathways	Limited standalone diagnostic performance; affected by sample quality, blood contamination, and inflammatory confounders	Auxiliary local inflammatory layer for combined diagnostic assessment rather than a definitive test

The diagnostic performance values summarized in this table are assay-, disease-, and study-context-specific estimates rather than universal parameters for all bone and joint infections. Reported sensitivity, specificity, AUC, and thresholds may vary according to disease entity, joint site, infection chronicity, organism virulence, prior antimicrobial exposure, specimen quality, assay platform, and diagnostic reference standard. Values are retained when supported by cited systematic reviews, meta-analyses, or clearly defined disease-specific studies; otherwise, qualitative evidence summaries and clinical positioning are provided.

### 3.2 Combined application of pathogen-derived metabolites and microenvironmental biochemical indicators

In addition to capturing host immune-response signals, direct detection of chemical traces left by pathogen metabolism provides another diagnostically specific strategy. Unlike immune molecules released by leukocytes, D-lactate is a stereospecific metabolite generated through glycolysis and fermentation by many major pathogenic bacteria, particularly *Staphylococcus* species and some members of the Enterobacteriaceae family, whereas mammalian cells produce only trace amounts of D-lactate and predominantly generate L-lactate (Li et al., 2025). The most common pathogens implicated in septic arthritis (SA) and periprosthetic joint infection (PJI) can produce measurable D-lactate in both planktonic and biofilm states, supporting its practical value as an indicator of bacterial or fungal infection (Morovic et al., 2024).A meta-analysis summarizing key studies in this field reported that synovial D-lactate showed a pooled sensitivity of 82%, specificity of 76%, diagnostic odds ratio of 14.18, and area under the receiver operating characteristic curve of 0.84 for diagnosing PJI. These findings indicate that D-lactate is a valuable synovial biomarker with favorable diagnostic performance and may serve as a reliable adjunctive biochemical indicator for rapid preoperative or intraoperative identification of PJI (Li et al., 2021).Further prospective cohort evidence has highlighted the diagnostic potential of combining synovial biochemical microenvironment indicators, including pH, D-lactate, and glucose. Extensive bacterial proliferation and active biofilm metabolism can rapidly consume free glucose in synovial fluid while releasing organic acid metabolites such as D-lactate, resulting in an acidic and low-glucose infected microenvironment. Clinical data showed that when the synovial D-lactate threshold was set at 8.45 mmol/L and combined with reduced synovial pH, this biochemical model achieved a negative predictive value of up to 84% (Müller et al., 2025). In addition, synovial D-lactate has shown diagnostic performance comparable to leukocyte count for identifying periprosthetic infection, while requiring only a small sample volume, short turnaround time, and low testing cost. Its sensitivity may also be associated with bacterial virulence (Yermak et al., 2019; Karbysheva et al., 2020). Therefore, this combined biochemical strategy provides a low-cost, rapidly deployable adjunctive approach for preoperative diagnosis, particularly in settings where advanced molecular platforms are not readily available.

## 4 Methodological advances in histopathology and microbiological culture as reference standards

### 4.1 Principles of multiple deep-tissue sampling and sonication

Despite rapid advances in molecular diagnostics and biomarker-based assays, deep-tissue sampling, microbiological culture, and histopathological assessment remain indispensable reference standards when determining whether to remove internal fixation devices, perform revision arthroplasty, or initiate long-term targeted antimicrobial therapy (Fonkoué et al., 2026). According to the joint recommendations of the Fracture-Related Infection (FRI) Consensus Group and the International Consensus Meeting (ICM), a key prerequisite for diagnosing bone- and implant-associated infections is the collection of at least three to five independent deep tissue or bone samples for rigorous microbiological analysis (Marais et al., 2023; Marais et al., 2024). In contrast, superficial swabs and sinus tract discharge smears, which were previously performed routinely in wards or outpatient settings, are highly susceptible to contamination by normal skin flora or hospital-associated resistant organisms. These specimens have low sensitivity and may provide misleading false-positive microbiological results, potentially leading to unnecessary use of broad-spectrum antibiotics (Khadka et al., 2025). In the clinical management of diabetic foot osteomyelitis (DFO), culture of bone biopsy specimens obtained directly from the infected bone margin remains an important reference standard. However, when percutaneous bone biopsy is not feasible because of anatomical risk or poor vascular conditions, deep-tissue culture may have alternative value under specific circumstances. A comparative study involving 107 patients with DFO showed that the overall microbiological concordance between deep-tissue culture and bone biopsy culture was 51.8%. For clinically common monomicrobial *Staphylococcus aureus* infection, the concordance of deep-tissue culture was the highest, reaching 44.4%. However, its reliability decreased substantially for Gram-negative bacteria and complex polymicrobial communities, highlighting the importance of directly obtaining deep bone matrix samples in resistant or polymicrobial infections (Ulusoy et al., 2025). In addition, the physical barrier formed by bacterial biofilms on implant surfaces has promoted the use of sonication fluid analysis to improve microbiological recovery. This technique uses low-frequency ultrasound at defined frequencies to generate physical vibration and cavitation in a sterile fluid containing removed joint prostheses, fracture fixation plates, screws, or other implants. The process disrupts and detaches biofilm structures densely adherent to metallic surfaces, resuspending embedded bacteria into a planktonic state. The resulting sonication fluid can then be concentrated by centrifugation and inoculated onto enriched culture media (Rothenberg et al., 2017). This physical processing step facilitates the release of slow-growing or low-virulence microorganisms hidden within biofilms, such as *Cutibacterium acnes* and coagulase-negative staphylococci, thereby reducing culture false-negativity.Beyond improving diagnostic yield by dislodging biofilm-associated microorganisms from removed arthroplasty components or fixation devices, sonication fluid can also provide host- and pathogen-derived proteins for further research. Such analyses may help characterize local host immune responses, clarify the pathophysiology of FRI, and identify genomic elements associated with infection. Accordingly, sonication has gradually become an important pre-analytical microbiological processing step in revision or implant-removal surgery for suspected implant-associated infection (Certain and Sigmund, 2025; O’Connor et al., 2025; Depypere et al., 2026).

### 4.2 Quantitative histopathological thresholds and differentiation from aseptic conditions

Histopathology provides important morphological evidence for bone and joint infections by directly visualizing acute inflammatory cell infiltration in infected bone tissue, periprosthetic membranes, and adjacent soft tissues. In recent years, the key progress in this field has not merely been the identification of inflammation, but rather the gradual establishment of more reproducible quantitative diagnostic criteria. However, the degree of inflammation required to define infection has long lacked a unified standard. Based on relevant histopathological validation studies, some criteria for acute inflammation in PJI and osteomyelitis have proposed thresholds based on the number of polymorphonuclear neutrophils (PMNs) observed across multiple high-power fields (HPFs, ×400 magnification), such as more than 23 PMNs per HPF in at least five HPFs (Bémer et al., 2018). For hip and knee periprosthetic joint infection (PJI), a 2025 systematic review and meta-analysis suggested that, when evaluated at ×400 magnification in the areas with the most prominent inflammation, ≥5 PMNs/HPF represents one of the most practical histological thresholds with optimal diagnostic performance. Under this criterion, the sensitivity was approximately 82.0%, the specificity was approximately 94.7%, and the area under the summary receiver operating characteristic curve (AUSROC) was approximately 0.963 (Sigmund et al., 2025b). It should be emphasized that histological thresholds are not fully interchangeable across different disease entities. For FRI and infected nonunion, current studies and consensus recommendations also support an average of >5 PMNs/HPF as important evidence suggestive of infection, whereas a complete absence of neutrophils is more consistent with aseptic nonunion (Stevenson et al., 2022). In contrast, biopsy specimens from spinal infections such as native vertebral osteomyelitis (NVO) often have a distinct inflammatory background. Their histological interpretation should therefore be integrated with microbiological findings and the clinical context, rather than mechanically applying PJI-derived criteria (Petri et al., 2024). Beyond neutrophil counting, a unique advantage of histopathology lies in its ability to provide spatial and structural information, including trabecular bone necrosis, bone marrow fibrosis, granuloma formation, foreign-body giant cell reactions, and chronic inflammatory cell infiltration. These morphological features have important supplementary value in distinguishing infectious lesions from aseptic loosening, wear particle-induced inflammatory reactions, and certain indolent low-virulence infections. Therefore, histopathology should not be viewed merely as a counting tool, but rather as an important morphological bridge linking host inflammatory responses with microbiological evidence (Peterlin et al., 2025; Sigmund et al., 2025a). Under microscopic examination, pathologists can identify not only neutrophilic infiltration, but also accompanying areas of bone matrix necrosis, granulomatous structures suggestive of atypical pathogens such as *Mycobacterium tuberculosis* or *Brucella* species, and chronic lymphocytic infiltration. This spatial structural analysis is essential for differentiating aseptic loosening caused by mechanical failure, foreign-body giant cell reactions, and low-virulence indolent infection (Kwon et al., 2017; Muñoz-Mahamud et al., 2026).

## 5 Targeted and untargeted molecular diagnostics in bone and joint infections

Molecular diagnostics in BJIs should not be regarded as a single uniform category. Clinical metagenomics and orthopedic infection reviews emphasize that targeted multiplex PCR panels, broad-range 16S rRNA gene PCR/qPCR, resistance-gene PCR assays, targeted amplicon sequencing, probe-capture or hybridization-enrichment NGS, and untargeted shotgun metagenomic sequencing differ substantially in target design, specimen requirements, analytical breadth, turnaround time, contamination risk, bioinformatic complexity, and clinical interpretability. In orthopedic infections, recent PJI-focused reviews and scoping reviews further support an algorithmic approach in which culture, mPCR, broad-range qPCR, tNGS, and mNGS are selected according to specimen type, clinical probability, culture status, and the need for antimicrobial resistance information. Therefore, diagnostic performance values derived from different molecular platforms should not be pooled or interpreted as interchangeable without methodological and disease-specific qualification (Chiu and Miller, 2019; Indelli et al., 2025; Kullar et al., 2025).

### 5.1 Broad-range 16S rRNA gene PCR/qPCR and targeted multiplex PCR panels

In clinical scenarios involving prior antimicrobial exposure, low pathogen burden, biofilm-embedded organisms, or slow-growing pathogens, conventional culture may show reduced diagnostic yield, prolonged turnaround time, or even negative results. Molecular assays, including multiplex PCR, broad-range PCR/sequencing, targeted NGS, and mNGS, can detect pathogen nucleic acids without requiring viable microbial growth and may therefore provide earlier etiological clues in culture-negative or microbiologically equivocal orthopedic infections. However, their major clinical value lies not in replacing culture, but in improving pathogen detection in selected difficult cases and supporting earlier antimicrobial decision-making together with culture, histopathology, specimen quality assessment, and clinical probability (Tang et al., 2021; Esteban et al., 2023; Kullar et al., 2023). Molecular diagnostic techniques that do not depend on the growth of viable microorganisms can directly detect pathogen nucleic acids and therefore have important adjunctive value in the etiological identification of complex bone and joint infections. However, the major clinical significance of molecular diagnostics lies not in completely replacing culture, but in shortening the time to etiological clues, improving pathogen detection in selected culture-negative cases, and supporting early antimicrobial decision-making together with subsequent culture-based verification. Broad-range or universal 16S rRNA gene PCR/qPCR is a targeted nucleic acid amplification strategy that uses conserved bacterial 16S rRNA gene primers to detect or quantify bacterial DNA in clinical specimens. However, unless followed by sequencing, this approach does not provide comprehensive species-level identification and should not be conflated with 16S rRNA gene amplicon sequencing or untargeted shotgun mNGS. Recent orthopedic infection reviews further emphasize that broad-range 16S detection may be most informative when paired with sequencing-based identification or integrated into a broader molecular diagnostic algorithm. Its main value is therefore as an adjunctive bacterial DNA detection tool rather than a comprehensive pathogen-identification platform (Kullar et al., 2025). Targeted multiplex PCR panels represent another rapid molecular strategy (Huang et al., 2025). These assays detect predefined pathogen and antimicrobial resistance gene targets within a closed workflow and can provide clinically actionable results within a short turnaround time. The BioFire Joint Infection Panel (BioFire JI Panel, BJIP) is a representative commercial example of this approach, but it is discussed here as one example of targeted multiplex PCR rather than as a preferred or exclusive diagnostic platform. This system uses a closed, sample-to-answer workflow and can complete nucleic acid extraction, amplification, and result interpretation within approximately one hour. The current panel covers 31 pathogen targets and 8 antimicrobial resistance gene targets, comprising 39 clinically relevant targets in total. These include common Gram-positive bacteria, Gram-negative bacteria, yeasts, and key resistance markers such as mecA/C and MREJ, vanA/B, KPC, NDM, OXA-48-like, VIM, IMP, and CTX-M (Esteban et al., 2023). The officially validated specimen type for the BioFire Joint Infection Panel is 0.2 mL of synovial fluid, and available evaluations have primarily focused on synovial fluid testing in suspected septic arthritis and PJI (Esteban et al., 2023; Saeed et al., 2023; Indelli et al., 2025). Although exploratory studies have investigated the BioFire Joint Infection Panel in non-synovial specimens, including bone, periarticular tissue, abscess drainage fluid, and other tissue samples from patients with suspected bone and joint infections, these applications remain outside the formally validated synovial fluid indication (Hoffman et al., 2023; Benvenuto et al., 2024). Therefore, disease entity and specimen type should be considered carefully when interpreting BJIP results, particularly in FRI, NVO, DFO, or non-implant-associated osteomyelitis, where diagnostic specimens and reference standards differ substantially from synovial fluid-based PJI testing. In a prospective study involving 151 patients with suspected septic arthritis or PJI, BJIP testing of synovial fluid showed a kappa agreement of 0.77 with conventional culture as the reference standard. For tissue biopsy specimens, the agreement was higher, reaching 0.80 (Salar-Vidal et al., 2026). In one prospective study, among patients who had received or were receiving antibiotics before sampling, the sensitivity of conventional culture decreased to approximately 35%, whereas BJIP showed a sensitivity of 68%, partly reflecting its ability to detect pathogen nucleic acids rather than relying on viable microbial growth. However, mPCR sensitivity varies substantially across studies; the Indelli et al., 2025 scoping review reported sensitivities ranging from 33% to 100% across mPCR studies, including BioFire-based evaluations, depending on population, specimen type, assay platform, antibiotic exposure, and reference standard (Indelli et al., 2025; Kaoual et al., 2025). These findings support the value of targeted multiplex PCR as a rapid adjunctive or salvage diagnostic tool in selected antibiotic-exposed cases. However, they should not be interpreted as evidence that multiplex PCR can replace culture or other reference methods. The interpretation of polymicrobial infection requires particular caution. Targeted multiplex PCR may rapidly detect selected on-panel co-pathogens and may identify some organisms that are difficult to recover by routine culture. Conversely, because multiplex PCR panels are restricted to predefined targets, they may miss off-panel organisms, including selected coagulase-negative staphylococci, uncommon anaerobes, slow-growing organisms, mycobacteria, fungi not included in the panel, or polymicrobial communities containing organisms outside the assay design. This limitation has been highlighted in BioFire-based studies and recent reviews, particularly in early postoperative or polymicrobial PJI, where clinically relevant organisms may fall outside the panel coverage (Schoenmakers et al., 2023; Gardete-Hartmann et al., 2024). In addition, at least one comparative BioFire Joint Infection Panel study reported that BJIP may underrepresent polymicrobial infection compared with conventional culture or broader sequencing-based approaches in some settings (Azad et al., 2022). Culture also has important limitations, including reduced sensitivity after antibiotic exposure, prolonged turnaround time, overgrowth of dominant organisms, and suboptimal recovery of anaerobes when sample collection, transport, or incubation conditions are not optimized. Therefore, the appropriate conclusion is not that targeted multiplex PCR is categorically superior to culture for polymicrobial infection, but that combined use of culture, targeted multiplex PCR, and, in selected difficult cases, sequencing-based assays may improve overall microbiological characterization. Implementation of BJIP in routine diagnostic workflows has been reported to shorten the turnaround time for pathogen and resistance-profile detection by an average of approximately 83 hours. This time advantage may enable clinicians to shift earlier from broad-spectrum empirical therapy to more targeted antimicrobial treatment and has been associated with optimization of early therapeutic strategies in up to 31% of infected patients (Schoenmakers et al., 2023). Overall, targeted multiplex PCR panels should be positioned as rapid adjunctive tools rather than replacements for conventional culture, histopathology, or disease-specific diagnostic criteria. Final etiological interpretation should integrate molecular results, culture findings, specimen type, off-panel limitations, clinical probability, and multidisciplinary assessment.

### 5.2 Untargeted shotgun metagenomic sequencing for broad pathogen detection

Sequencing-based molecular approaches provide an important extension beyond closed multiplex PCR panels, especially in culture-negative, polymicrobial, low-burden, or antibiotic-exposed BJIs. However, these methods should not be treated as a single diagnostic category. Targeted amplicon-based sequencing, probe-capture or hybridization-enrichment NGS, and untargeted shotgun metagenomic sequencing differ substantially in primer or probe dependence, analytical breadth, host-background interference, contamination risk, bioinformatic complexity, and clinical interpretability (Olearo et al., 2025; Salar-Vidal et al., 2026; Tipton et al., 2026). Therefore, separating targeted and untargeted NGS approaches is necessary for accurately interpreting their diagnostic value across PJI, FRI, NVO, DFO, and other osteomyelitis-related conditions.

#### 5.2.1 Targeted NGS-based approaches: 16S/ITS amplicon sequencing and probe-capture methods

Targeted NGS-based approaches still rely on predefined primers or enrichment probes and should therefore be distinguished from untargeted shotgun mNGS. This distinction is particularly important in orthopedic infection diagnostics, where broad-range PCR/qPCR, targeted sequencing, and mNGS may serve different roles depending on specimen type, culture status, and the required level of pathogen identification (Kullar et al., 2025). This approach differs from broad-range 16S rRNA gene PCR/qPCR, which detects or quantifies bacterial DNA but does not provide comprehensive species-level identification unless followed by sequencing. In culture-negative BJIs, 16S rRNA gene amplicon sequencing may be useful when conventional culture is compromised by prior antibiotic exposure, low bacterial burden, fastidious organisms, or biofilm-associated growth restriction (Olearo et al., 2025; Salar-Vidal et al., 2026). However, it remains limited to bacterial detection, is affected by primer bias and database quality, and usually provides limited information on antimicrobial resistance unless additional resistance-gene assays are performed. For suspected fungal infection, ITS amplicon sequencing provides a parallel targeted sequencing strategy by amplifying fungal internal transcribed spacer regions. Its clinical role in BJIs remains less standardized than bacterial 16S-based approaches and is mainly relevant in selected immunocompromised patients, chronic indolent infections, or cases with strong clinical suspicion of fungal involvement (Kullar et al., 2025; Tipton et al., 2026). Probe-capture or hybridization-enrichment NGS represents another semi-targeted strategy. In this workflow, predefined hybridization probes are used to enrich selected microbial sequences or resistance determinants from sequencing libraries before downstream analysis. This approach may improve detection of predefined targets and reduce sequencing burden, but it remains dependent on panel design and may introduce target bias, additional processing steps, higher cost, and longer turnaround time. Although probe capture-based tNGS has been explored in other infectious disease settings, its orthopedic infection-specific evidence remains less mature than that for 16S amplicon-based tNGS or shotgun mNGS (Chiu and Miller, 2019; Hong et al., 2023; Cai et al., 2024). Overall, targeted NGS-based approaches occupy an intermediate position between conventional PCR and untargeted shotgun mNGS. They may offer improved taxonomic resolution compared with simple broad-range PCR and lower host-background interference than shotgun mNGS, but they are not fully unbiased. Their negative results should therefore be interpreted according to primer or probe coverage, specimen type, microbial burden, prior antimicrobial exposure, and disease-specific validation. In PJI and selected FRI cases, targeted NGS may be particularly useful as an adjunctive tool for culture-negative infection, whereas evidence in NVO, DFO, and non-implant-associated osteomyelitis remains more dependent on biopsy quality and local validation.

#### 5.2.2 Untargeted shotgun metagenomic sequencing for broad pathogen detection

When used in the strict sense of untargeted shotgun metagenomic sequencing, mNGS does not rely on organism-specific or universal amplicon primers. Instead, it sequences total nucleic acids extracted from clinical specimens and identifies microbial sequences through bioinformatic comparison with reference databases (Zhao et al., 2024). This definition excludes 16S rRNA gene amplicon sequencing, ITS amplicon sequencing, and probe-capture NGS, all of which rely on predefined primer pairs or enrichment probes and should therefore be discussed separately from shotgun mNGS. Compared with targeted NGS-based methods, untargeted shotgun mNGS has broader pathogen-discovery potential because it can theoretically detect bacteria, fungi, viruses, and uncommon or unexpected organisms within a single workflow. In clinical bone tissue, synovial fluid, sonication fluid, or abscess specimens, shotgun mNGS can identify microbial nucleic acid fragments without requiring viable growth in culture. This may be particularly valuable in culture-negative BJIs, polymicrobial infections, low-virulence infections, infections after prior antimicrobial exposure, and diagnostically complex biopsy-based cases such as NVO (Huang et al., 2023; Schoenmakers et al., 2023; Zhao et al., 2024).However, the evidence base for sequencing-based assays should be interpreted cautiously. Some meta-analyses and clinical studies report diagnostic performance under the broad NGS/mNGS umbrella, but included methods may involve both targeted amplicon-based sequencing and untargeted shotgun metagenomic sequencing. Therefore, pooled sensitivity and specificity values should not be interpreted as the performance of shotgun mNGS alone (Schoenmakers et al., 2023; Olearo et al., 2025). In addition, the broad analytical scope of shotgun mNGS introduces important interpretive challenges. Host DNA background, environmental contamination, skin commensals introduced during sampling, database misclassification, residual DNA from nonviable organisms, and inconsistent reporting thresholds can all affect clinical specificity (Zhang et al., 2023; Zhao et al., 2024; Lin et al., 2025; Zhang et al., 2025; Zhou et al., 2025). Therefore, shotgun mNGS should not be used as an isolated “report-to-prescription” tool. Sequencing read counts, genome coverage, taxonomic plausibility, specimen type, prior antibiotic exposure, culture results, histopathology, imaging findings, and the patient’s clinical course should be interpreted together within an MDT framework. In this sense, shotgun mNGS is best positioned as an advanced adjunctive tool for selected difficult cases rather than a universal replacement for culture, histopathology, or disease-specific diagnostic criteria. (**Table 3**).

**Table 3:** Methodological distinction among nucleic acid-based assays used in BJI diagnosis.

Method	Target design	Main output	Strength	Main limitation
Broad-range 16S rRNA gene PCR/qPCR	Universal bacterial primers	Bacterial DNA detection or quantification	Simple and broad bacterial screening	Limited species resolution unless sequenced; no fungal/viral detection
16S rRNA gene amplicon sequencing	Universal bacterial primers followed by sequencing	Bacterial taxonomic identification	Useful for culture-negative and selected polymicrobial bacterial infections; less costly and less data-intensive than untargeted shotgun mNGS	Primer bias; limited to bacteria; not untargeted
ITS amplicon sequencing	Fungal ITS primers	Fungal taxonomic identification	Useful for fungal detection	Fungal-focused; primer and database limitations
Targeted multiplex PCR	Predefined pathogen/resistance targets	Rapid detection of selected organisms and genes	Fast and clinically actionable	Cannot detect organisms outside the panel
Probe-capture NGS	Predefined enrichment probes	Enriched microbial sequencing	Useful for culture-negative and selected polymicrobial infections when predefined targets are covered; less data-intensive than untargeted shotgun mNGS	Semi-targeted; depends on probe design
Untargeted shotgun mNGS	No predefined amplicon primers	Broad microbial sequence detection	Broadest pathogen discovery potential	Host background, contamination, cost, bioinformatic complexity

### 5.3 VBNC state: unique value of molecular technologies in culture-negative infection

In orthopedic implant-associated infections, a substantial proportion of apparently aseptic or culture-negative cases may be closely related to bacterial biofilm formation and occult pathogens entering a viable but non-culturable (VBNC) state (Šuster and Cör, 2023; Liu et al., 2023). When common implant-associated pathogens, such as Staphylococcus species and Pseudomonas aeruginosa, encounter severe nutrient deprivation and hypoxia within deep biofilm layers or are exposed to strong antimicrobial pressure from agents such as vancomycin or gentamicin, they may not be directly eradicated. Instead, they can activate survival stress responses and upregulate key genes such as codY and pdhA. This genetic reprogramming alters bacterial glucose metabolism and drives the organisms into a deeply dormant state characterized by extremely low metabolic activity (Gaio et al., 2021). In this state, bacteria no longer undergo active cell division and therefore lose the ability to form colonies on standard laboratory agar media. After entering the VBNC state under antimicrobial pressure, *Staphylococcus aureus* can reportedly survive and maintain low-level gene expression within biofilms for up to 150 days. Once antibiotics are discontinued, host immunity declines, or specific metabolic intermediates such as sodium pyruvate become available, these VBNC bacteria may resuscitate, regain virulence, and contribute to recurrent or persistent infection (Pasquaroli et al., 2013). In this context, conventional culture is inherently limited. Molecular nucleic acid-based technologies, including mNGS and quantitative PCR (qPCR), combined with transcriptomic assays targeting RNA markers of transcriptional activity, may help detect residual pathogen-derived nucleic acids and transcripts despite the absence of culture growth. Such approaches may provide an effective laboratory strategy for identifying VBNC-associated culture-negative infections and clarifying otherwise hidden infectious processes. (**Tables 4**, **5**).

**Table 4:** Disease-specific applicability of major laboratory diagnostic approaches in bone and joint infections.

Diagnostic approach	PJI	FRI	NVO	DFO/osteomyelitis	Main limitation
CRP/ESR	Screening/rule-out adjunct	Screening adjunct; limited specificity after trauma	Useful for suspicion and monitoring	ESR may support screening in DFO	Nonspecific systemic inflammation
NLR and blood-count indices	Emerging adjunct	Emerging screening/risk marker	Limited evidence	Limited evidence	Cut-offs not standardized
Alpha-defensin	Strongest evidence in synovial fluid	Limited/emerging	Not directly applicable	Not directly applicable	Requires appropriate joint fluid
Calprotectin	Strong evidence in PJI	Emerging but not standardized	Not directly applicable	Not standardized	Disease-specific thresholds lacking
Deep-tissue culture	Important in revision surgery	Core diagnostic method	CT-guided biopsy when blood cultures are negative	Bone biopsy preferred	Sampling quality and antibiotics affect yield
Histopathology	Established adjunct	Important confirmatory/suggestive criterion	Supportive with biopsy	Useful for bone involvement	Thresholds differ by disease entity
Sonication	Useful for removed implants	Useful for implant-associated FRI	Limited	Limited	Requires removed hardware
BioFire Joint Infection Panel	Validated mainly for synovial fluid	Exploratory/non-validated samples	Not standard	Not standard	Limited to predefined targets
mNGS	Useful in culture-negative or polymicrobial cases	Useful but interpretation challenging	Useful in selected biopsy-based cases	Useful in selected refractory cases	Contamination and reporting thresholds
AI/ML models	Emerging PJI models	Early FRI models	Limited	Limited	External validation lacking

**Table 5:** Contextualized interpretation of microbiological, histopathological, and molecular assays in bone and joint infections.

Diagnostic method	Sample source	Applicable scenarios	Evidence summary/key parameters	Main advantages	Main limitations	Clinical positioning
Multiple deep-tissue culture	Deep bone/soft-tissue samples	Intraoperative etiological diagnosis of FRI, PJI, DFO, and osteomyelitis	Consensus recommendations support collection of at least 3–5 independent deep-tissue samples for microbiological analysis	Remains a key etiological reference and can guide targeted antimicrobial therapy.	Affected by preoperative antibiotics, biofilm formation, low-virulence organisms, transport conditions, incubation protocols, and sampling quality.	Standard intraoperative microbiological foundation; should be optimized before relying on adjunctive molecular testing.
Bone biopsy culture	Infected bone tissue	DFO and deep bone infections	Bone biopsy culture remains an important reference standard in DFO and selected deep bone infections, although feasibility varies by anatomical site and patient condition	More closely reflects the true intraosseous pathogen profile than superficial swabs.	Percutaneous biopsy may be limited by anatomical risk, vascular status, patient tolerance, and prior antimicrobial exposure.	High-value sampling approach for deep bone infection, especially when superficial cultures are unreliable.
Sonication fluid culture	Removed prostheses/internal fixation devices	Implant-associated infection and revision surgery	Sonication can improve recovery of biofilm-associated organisms from removed implants and is most relevant when prostheses or fixation devices are available for processing	Improves detection of slow-growing, low-virulence, and biofilm-associated organisms.	Applicable only when implants are removed; requires standardized processing and careful contamination control.	Important yield-enhancing method for implant-associated infection rather than a standalone diagnostic replacement.
Histopathology	Deep tissue/periprosthetic membrane	PJI, FRI, infected nonunion, and related conditions	In PJI, meta-analytic evidence supports ≥5 PMNs/HPF at ×400 magnification as a practical threshold with high specificity; however, thresholds and performance vary across disease entities and sampling sites	Provides direct morphological evidence of infection and identifies structural features such as bone necrosis, granulomas, chronic inflammation, and foreign-body reactions.	PJI-derived thresholds should not be mechanically applied to NVO, DFO, or all osteomyelitis contexts; interpretation depends on tissue quality and pathological expertise.	Important morphological adjudication tool that complements microbiology and molecular findings.
Broad-range 16S rRNA gene PCR/qPCR	Synovial fluid, sonication fluid, deep tissue, or bone biopsy specimens	Culture-negative PJI, FRI, NVO, DFO, and other suspected bacterial BJIs	Detects or quantifies bacterial DNA using conserved 16S rRNA gene primers; species-level identification usually requires subsequent sequencing or integration into a broader molecular workflow	Does not require viable bacterial growth; useful as an adjunct when culture is negative or antibiotic exposure is present.	Does not provide comprehensive species-level identification unless followed by sequencing; no direct phenotypic susceptibility information; contamination may cause false-positive results.	Adjunctive bacterial DNA detection method; should not be conflated with 16S rRNA gene amplicon sequencing, multiplex PCR panels, or untargeted shotgun mNGS.
Targeted multiplex PCR panels, e.g., BioFire Joint Infection Panel	Formally validated mainly for synovial fluid; exploratory use in tissue or other specimens requires caution	Primarily PJI and septic arthritis; selected complex cases after antibiotic exposure	Rapid detection of predefined pathogen targets and selected resistance genes. BioFire JI Panel reports results within approximately 1 hour and includes 31 pathogen targets plus 8 resistance gene targets; reported mPCR sensitivity varies widely across studies, including BioFire-based evaluations	Rapid closed workflow; simultaneous detection of selected organisms and resistance markers; useful for early antimicrobial decision support.	Limited to predefined targets; may miss off-panel organisms, uncommon anaerobes, mycobacteria, fungi not included in the panel, or polymicrobial communities containing off-panel pathogens.	Rapid targeted molecular adjunct; best interpreted together with culture, specimen type, off-panel limitations, clinical probability, and MDT assessment.
16S/ITS amplicon-based targeted NGS	Synovial fluid, tissue, sonication fluid, or biopsy specimens depending on platform and validation	Culture-negative or antibiotic-exposed infections; selected PJI and FRI cases; fungal infection when ITS is included	Uses predefined bacterial 16S rRNA and/or fungal ITS primers followed by sequencing. Some commercial amplicon-based targeted NGS panels combine bacterial 16S and fungal ITS profiling from the same specimen	Provides higher taxonomic resolution than simple broad-range PCR; can detect bacteria and, when ITS is included, fungi.	Primer bias, limited antimicrobial resistance information, contamination risk, database dependence, and variable disease-specific validation.	Intermediate targeted sequencing approach; useful in selected culture-negative cases but not equivalent to untargeted shotgun mNGS.
Probe-capture/hybridization-enrichment NGS	Platform-dependent clinical specimens	Selected infections requiring enrichment of predefined microbial or resistance targets	Uses predefined hybridization probes to enrich selected microbial sequences or resistance determinants before sequencing. Orthopedic infection-specific evidence remains less mature than that for amplicon-based tNGS or shotgun mNGS	May improve detection of predefined targets and reduce sequencing burden.	Still panel-dependent; may introduce target bias, additional processing steps, higher cost, and longer turnaround time.	Semi-targeted molecular strategy; should be described as exploratory or platform-dependent in BJIs unless orthopedic-specific validation is available.
Untargeted shotgun mNGS	Synovial fluid, bone tissue, sonication fluid, abscess material, or deep biopsy specimens	Culture-negative infection, low-virulence organisms, polymicrobial infection, unusual pathogens, and complex recurrent cases	Broad NGS/mNGS studies report favorable diagnostic potential, but pooled estimates may combine targeted amplicon sequencing, capture-based approaches, and shotgun metagenomics; therefore, values should not be interpreted as the fixed performance of shotgun mNGS alone	Broad pathogen-detection potential without predefined organism-specific primers; useful for rare, mixed, biofilm-associated, or unexpected pathogens.	Susceptible to contamination, host DNA background, database misclassification, residual nonviable microbial DNA, inconsistent reporting thresholds, and limited standardization.	Advanced adjunctive molecular test for selected difficult cases; results require interpretation with culture, histopathology, imaging, specimen quality, and clinical context.

This table summarizes disease- and method-contextualized evidence rather than universal diagnostic performance. For culture, histopathology, and molecular assays, reported sensitivity, specificity, turnaround time, and diagnostic yield may vary according to specimen source, infection entity, pathogen burden, biofilm status, prior antimicrobial exposure, assay platform, contamination-control procedures, and diagnostic reference standard. Single-point performance estimates were retained only when they were clearly linked to a cited disease-specific study or meta-analysis; otherwise, qualitative evidence summaries and clinical positioning are provided.

## 6 Systems biology of host–pathogen interactions: integration of omics and microecological approaches

### 6.1 Host immune proteomic features and antibody targets in PJI

Immune proteomics aims to use ultra-high-resolution mass spectrometry to map the broad spectrum of cytokines, chemokines, antibodies, and cascade-associated signaling molecules synthesized and released by immune cells at different levels in response to microbial invasion by specific antigenic pathogens (Magruder et al., 2026). Using liquid chromatography–tandem mass spectrometry (LC-MS/MS) and high-throughput multiplex array platforms, such as Simoa-based technologies, recent studies have applied the Olink Proteomics 92-protein inflammation panel to sonication fluid samples from patients with non-infectious arthroplasty failure (NIAF) and PJI. Proteomic analysis of 200 sonication fluid samples has shown that differential expression patterns of inflammatory proteins can distinguish PJI from NIAF and provide deeper insight into immune responses during arthroplasty failure. Such sonication fluid-based proteomic profiling may help differentiate PJI from non-infectious arthroplasty failure and may further support the classification of clinical subtypes within PJI and/or NIAF (Cr et al., 2022). Proteomic studies suggest that the dominant host response in PJI is often characterized by the upregulation of antimicrobial and inflammatory proteins (Magruder et al., 2026). Consistent findings have shown that proteinase 3 (PRTN3), myeloid cell nuclear differentiation antigen (MNDA), the bacteriostatic protein lactoferrin (LTF), and a series of complement activation proteins, including C1q, C3b/C3i, and C5a, are markedly upregulated in local infected specimens from patients with PJI, such as synovial fluid and periprosthetic bone tissue. In contrast, these proteins are expressed at very low levels or remain largely silent in aseptic samples associated with mechanical loosening (Wang et al., 2019; Fröschen et al., 2021). Analysis of antibody titers against pathogen-specific antigens generated during infection has also shown clinical value beyond conventional diagnosis, particularly for prognostic stratification. For example, in humoral immune studies of *Staphylococcus aureus* bone infections, including PJI, high serum IgG titers against the heme-scavenging protein IsdB have been associated with an increased risk of poor clinical outcomes, including arthrodesis, reinfection, amputation, and death due to sepsis (Nishitani et al., 2020; Muthukrishnan et al., 2021). Conversely, high IgG titers against the glucosaminidase Gmd, a subunit of the autolysin Atl, and other autolysin-related antigens appear to be associated with protective immune responses and a reduced risk of postoperative adverse events, with each 10-fold increase in antibody concentration corresponding to an approximately 60% reduction in adverse-event risk (Kates et al., 2020). These findings extend the role of laboratory testing from merely determining the presence or absence of infection to supporting prognostic stratification and individualized therapeutic decision-making.

### 6.2 Host-response transcriptomics for highly sensitive detection of early immune activation

Transcriptomics enables high-throughput capture and analysis of messenger RNA (mRNA) expression profiles in peripheral blood or affected local tissues. By doing so, it can sensitively detect early immune pathway activation before substantial protein synthesis occurs or overt clinical manifestations become evident (Feng et al., 2025). In several bone biopsy specimens from patients with suspected fracture-related infection (FRI), where conventional culture was negative and clinical presentation was equivocal, deep transcriptomic analysis not only detected bacterial transcripts through low-abundance sequence alignment, but also revealed specific activation of chemokine signaling pathways in the host tissue matrix, including CXCL1, CXCL2, and CCL4/L1/L2 (Depypere et al., 2026). These findings suggest that transcriptomic signals may shift infection detection upstream to the level of gene transcription. For broader systemic acute infections and the risk of sepsis-related bloodstream infection, molecular host-response platforms such as TriVerity™, which is based on a 29-mRNA marker panel, have shown potential advantages over combinations of conventional single protein biomarkers such as PCT and CRP in challenging emergency department settings. These platforms may help distinguish severe bacterial infection from viral infection and predict the risk of rapid clinical deterioration. Early severity assessment in adults with suspected infection, together with timely consideration of bacterial or viral etiologies, may reduce unnecessary laboratory testing and emergency care resource utilization. Such multiplex mRNA-based platforms are being increasingly explored as rapid clinical assessment tools for severe bone and joint infections complicated by systemic sepsis or bloodstream infection (Wu et al., 2025).

### 6.3 Local and gut microbiome dysbiosis and the infection axis

Implant-associated infections were traditionally considered to arise mainly from exogenous contamination of the surgical wound. However, with advances in human microbiome research, the orthopedic field has increasingly recognized that the long-term balance of the host microecosystem, particularly the gut microbiota, plays a key role in regulating systemic immune defense thresholds (Heckmann et al., 2025). Gut microbiome dysbiosis induced by prolonged antibiotic use, metabolic diseases such as obesity and diabetes, or inflammatory bowel disease may impair systemic immune homeostasis and may also serve as a hidden reservoir for endogenous pathogens such as *Staphylococcus aureus* (Ramasamy et al., 2025). Under the stress of surgical trauma, these microorganisms may translocate through a so-called “Trojan horse” mechanism. Increased intestinal permeability may allow bacteria and their metabolites to disseminate through the bloodstream or lymphatic circulation and subsequently colonize metallic prostheses or internal fixation materials, where they can form biofilms. Landmark animal model studies have shown that antibiotic-induced disruption of the gut microbiota significantly increases the incidence of PJI in mice, accompanied by reduced splenic immune cell populations and weakened systemic inflammatory responses, further supporting a direct contribution of dysbiosis to local infection susceptibility (Cj et al., 2019). Clinically, patients with PJI have been reported to show significantly higher serum zonulin and soluble CD14 (sCD14) levels than non-infected controls, with even greater increases in acute infection. Patients with inflammatory bowel disease also show a higher cumulative incidence of PJI after total joint arthroplasty (Chisari et al., 2022). A deeper understanding of this complex gut–bone immune axis, sometimes referred to as osteomicrobiology, may not only refine the mechanistic framework of BJI/PJI pathogenesis, but also provide new laboratory research directions and translational opportunities. In the future, perioperative strategies aimed at restoring host microecological balance, such as targeted probiotic or microbiome-modulating interventions, may contribute to the prevention of BJIs, although robust clinical validation remains necessary (Sharma et al., 2024).

## 7 From composite diagnostic definitions to artificial intelligence and machine learning in clinical diagnostic decision-making

### 7.1 Existing composite diagnostic definitions as the clinical foundation for data integration

Before discussing AI- or ML-based diagnostic models, it is important to recognize that the laboratory diagnosis of BJIs has already moved from single-test interpretation toward composite diagnostic frameworks. In PJI, this transition is particularly advanced. The MSIS and ICM definitions combine major diagnostic criteria, such as a sinus tract communicating with the prosthesis or repeated isolation of the same pathogen from separate specimens, with multiple minor criteria derived from serum inflammatory markers, synovial fluid analysis, microbiology, histopathology, and intraoperative findings (Workgroup Convened by the Musculoskeletal Infection Society, 2011; Parvizi et al., 2018). The 2018 ICM definition further formalized this approach through a weighted scoring system, thereby reflecting the principle that no single biomarker can reliably define infection across all clinical settings (Parvizi et al., 2018). The EBJIS definition provides another clinically practical framework by classifying suspected cases into infection unlikely, infection likely, and infection confirmed, which is particularly useful for managing borderline or inconclusive cases (McNally et al., 2021). In FRI, composite diagnosis is also used, but the framework is organized differently. Rather than relying on a weighted numerical score, the FRI consensus definition separates diagnostic features into confirmatory and suggestive criteria. Confirmatory criteria, such as a sinus tract or wound breakdown communicating with bone or implant, purulence, phenotypically indistinguishable pathogens from at least two separate deep samples, or microorganisms demonstrated by histopathology, indicate that infection is definitely present (Metsemakers et al., 2018; Govaert et al., 2020). Suggestive criteria, including local inflammatory signs, fever, persistent wound drainage, radiological abnormalities, elevated serum inflammatory markers, or a single positive culture, should prompt further investigation but are not sufficient alone to confirm infection (Metsemakers et al., 2018; Govaert et al., 2020). This distinction is clinically important because FRI often occurs in the setting of trauma, fracture healing, soft-tissue injury, and implant fixation, where systemic inflammatory markers and imaging findings may be less specific than in elective arthroplasty settings. For NVO, DFO, and other forms of osteomyelitis, composite diagnostic pathways are generally less standardized than those used for PJI. Diagnosis usually depends on integrating clinical presentation, serial inflammatory markers, imaging, blood culture, image-guided or surgical biopsy, microbiological culture, molecular testing when appropriate, and histopathological interpretation (Berbari et al., 2015; Senneville et al., 2024). Therefore, the current field can be viewed as progressing along a continuum: PJI has the most mature composite scoring and classification systems; FRI has a consensus-based confirmatory/suggestive framework; and NVO, DFO, and broader osteomyelitis still rely more heavily on disease-specific multidisciplinary interpretation. AI- and ML-based models should therefore be understood not as replacements for these established definitions, but as potential extensions of composite diagnosis that may help integrate larger, more heterogeneous, and more data-rich diagnostic variables.

### 7.2 Development and application of algorithm-driven integrated diagnostic prediction models

Building on these composite diagnostic frameworks, AI and ML approaches aim to extend conventional multi-criteria interpretation into higher-dimensional data integration. With the increasing availability of serum biochemical indicators, synovial molecular biomarkers, microbiological and histopathological results, high-throughput omics data, clinical susceptibility factors, and complex imaging features, the dimensionality of diagnostic data generated from a single patient has increased substantially. These high-dimensional heterogeneous data are often large in volume, interrelated, and noisy, making comprehensive interpretation increasingly challenging when relying solely on conventional experience-based clinical reasoning. In this context, artificial intelligence (AI) and machine learning (ML) frameworks provide powerful computational tools for integrating multimodal data and reducing diagnostic uncertainty in complex infections, thereby supporting precision medicine (Albano et al., 2023; Nairne‐Nagy et al., 2026). Clinically, many patients with fracture-related infection (FRI) do not present with obvious sinus tracts, fever, or purulent discharge. Instead, their clinical manifestations may be subtle and nonspecific, making them prone to misdiagnosis as ordinary fracture nonunion. To address this challenge, researchers developed the FRID-PE nomogram, an ML-based model for early FRI prediction, using high-resolution ^18F-FDG PET/CT imaging data combined with laboratory biomarkers. This model incorporated least absolute shrinkage and selection operator (LASSO) regression and multivariable Cox proportional hazards analysis (Yang et al., 2025). It integrated local imaging-based molecular metabolic features with three core systemic inflammatory laboratory indicators: the systemic immune-inflammation index (SII), IL-6, and ESR. In cross-validation, the model showed favorable diagnostic performance, and decision-curve analysis suggested that model-guided clinical intervention could provide greater net clinical benefit than uniformly applying either aggressive or conservative strategies to all patients. In the field of PJI diagnosis, unsupervised machine learning has been used to perform deep clustering of expression patterns across 11 synovial biomarkers, including specimen integrity markers such as A280 and synovial fluid red blood cell count (SF-RBC), inflammatory markers such as WBC count, percentage of neutrophils, and synovial fluid CRP, In the field of PJI diagnosis, unsupervised machine learning has been used to perform deep clustering of expression patterns across 11 synovial biomarkers, including specimen integrity markers such as A280 and synovial fluid red blood cell count, inflammatory markers such as WBC count, percentage of neutrophils, and synovial fluid CRP, PJI-associated host-response markers such as alpha-defensin, and selected pathogen antigen markers, as introduced above as local pathogen-directed immunoassays. This approach led to the development of a comprehensive probability-based PJI diagnostic scoring system, SynTuition™ (Parr et al., 2025; Parr et al., 2026). A key feature of this model is that it does not rely on delayed or potentially false-negative bacterial culture results as input. Instead, it uses parallel multi-biomarker profiling to support infection versus non-infection classification within 24 hours, particularly in diagnostically challenging culture-negative cases or cases with borderline biochemical results. This strategy may help reduce ambiguity in the real-world application of conventional ICM criteria and improve diagnostic consistency.

### 7.3 Innovative integration of isothermal microcalorimetry and deep learning

Isothermal microcalorimetry (IMC) is a highly sensitive biophysical detection method that can continuously and non-destructively capture subtle heat-flow changes generated by microbial metabolism and proliferation in synovial fluid samples, thereby producing characteristic thermal activity curves (Alvarez-Lorenzo and Concheiro, 2025). However, the heat-flow curves generated by different bacterial species may be highly similar, making species-level differentiation difficult using visual inspection or conventional statistical methods alone. In this context, computer vision and advanced AI approaches have been introduced to enhance IMC-based interpretation. In a representative analysis of 413 heat-flow curves derived from clinical samples, an XGBoost-based binary classification model achieved 100% detection accuracy for identifying whether synovial fluid samples were associated with PJI. For the more challenging task of species-level pathogen differentiation, which is difficult for IMC alone, an XGBoost multiclass model and a combined convolutional neural network plus multilayer perceptron (CNN+MLP) model achieved identification accuracies of 90.3% and 91.5%, respectively (Lozano‐García et al., 2025). Earlier validation studies of IMC further showed that, compared with conventional culture, IMC increased sensitivity from 69% to 83%, while both methods maintained 100% specificity. Moreover, the median time to detection (TTD) was shortened from 51 hours to 10 hours; even in the subgroup with chronic antibiotic exposure, IMC retained a sensitivity of 93% (Cichos et al., 2023). In the field of fracture-related infection (FRI), IMC has also demonstrated notable time-saving advantages. In addition, IMC can detect persister cells within biofilms, providing a useful tool for mechanistic studies of difficult-to-treat infections (Butini et al., 2019; Cichos et al., 2022). The integration of AI with IMC may therefore enhance the clinical interpretability of metabolic heat-flow signals and provide a rapid pathogen-detection strategy that does not rely on predefined molecular targets. This approach has the potential to support infection classification and pathogen-level identification within 24 hours, thereby informing individualized antibiotic selection and decontamination strategies, although further multicenter validation and workflow standardization remain necessary before routine clinical implementation (Lozano‐García et al., 2025).

### 7.4 Validation limitations and standardization challenges of AI models

Although AI-assisted models have shown considerable clinical potential in various validation settings, their large-scale real-world deployment and regulatory approval still face substantial limitations. Currently published AI-based prediction models for orthopedic infections, particularly those focused on periprosthetic joint infection (PJI), are mostly derived from specific regions, single-center settings, and retrospective cohorts, and often lack rigorous prospective and multicenter external validation (Vulpe et al., 2025). Bias in medical AI can arise at multiple stages, including data characteristics, labeling, model development, evaluation, and deployment. Uneven sample distribution and insufficient population representativeness may lead to reduced performance in certain patient subgroups (Cross et al., 2024). Therefore, the generalizability of these models across institutions, geographic regions, and patient populations remains to be further confirmed. Heterogeneity among medical institutions in case composition, laboratory workflows, data acquisition methods, and equipment platforms, together with potential demographic bias in training datasets, may all affect model robustness and transferability. Clinical prediction models should improve transparency in reporting and validation quality to support real-world translation (Collins et al., 2024; Rockenschaub et al., 2024). In addition, many deep learning or ensemble models still retain a degree of “black-box” opacity, and the limited interpretability of their decision-making processes may reduce clinicians’ trust and acceptance, especially in high-risk medical decision-making. Although general generative large language models based on natural language processing (NLP) have shown some utility in assisting medical record extraction, they currently do not have sufficient medical reliability to directly guide targeted antibiotic strategies or make complex clinical diagnostic decisions. Their use should therefore be strictly limited to educational support and literature assistance rather than direct clinical decision-making (Zellner et al., 2025). By contrast, AI-based computer-aided diagnosis (CAD) systems have shown potential in the diagnosis and grading of orthopedic-related diseases. These models may serve as decision-support tools by providing consistent preliminary grading, helping junior radiologists and medical trainees reduce interpretive variability and improve confidence in early diagnosis (Shahid et al., 2025).The next stage of AI development in orthopedic infection will depend heavily on the construction of globally standardized, multimodal, open-access datasets, as well as the development of interpretable AI algorithmic frameworks that meet stringent medical device regulatory requirements.

## 8 Reconstructing special infection pathways: NVO standardization and multidisciplinary collaboration

### 8.1 Integrating the definition of native vertebral osteomyelitis and addressing disease heterogeneity

The value of precise laboratory diagnosis lies not only in advances in individual diagnostic technologies, but also in whether these data can be effectively incorporated into standardized disease-management frameworks and seamlessly connected multidisciplinary care models. International orthopedic organizations, including the International Consensus Meeting (ICM), have increasingly emphasized the need to reduce diagnostic heterogeneity and treatment delays caused by inconsistent disease definitions and specialty barriers (Oral et al., 2026). Native vertebral osteomyelitis (NVO) accounts for approximately 3%–5% of all osteomyelitis cases and is often insidious in onset. Patients commonly present with persistent, nonspecific lower back pain as the initial or only symptom, and many lack typical systemic signs of infection, such as fever, during the early stage (Wa et al., 2026). This nonspecific presentation may lead to diagnostic delays lasting several months. If the causative pathogen is not identified and appropriate treatment is not initiated in a timely manner, irreversible spinal cord compression, paralysis, or even death may occur. Historically, the lack of unified terminology for NVO, which has often been used interchangeably with discitis or spondylitis, and the absence of standardized diagnostic definitions have generated substantial heterogeneity among studies evaluating mortality, neurological recovery, and infection recurrence after antimicrobial therapy (Petri et al., 2024; Zhu et al., 2024). To address this problem, Petri and colleagues proposed an integrated diagnostic framework for NVO. This framework reorganizes previously fragmented diagnostic approaches and converges NVO diagnosis into three core domains: clinical assessment, radiological imaging, and direct microbiological evidence. These domains are further divided into seven practical categories: (a) detailed clinical examination findings; (b) dynamic changes in systemic inflammatory biomarkers; (c) imaging techniques such as high-resolution magnetic resonance imaging (MRI); (d) serial blood culture microbiology; (e) computed tomography (CT)-guided spinal biopsy; (f) deep tissue histopathological assessment; and (g) monitoring of early clinical response to empirical or targeted antimicrobial therapy (Petri et al., 2024). This multidimensional framework moves away from premature diagnostic judgment based on a single imaging result or one-time culture result, and is being further refined through a multi-round Delphi consensus process involving international experts. However, definitional standardization does not imply that NVO is a microbiologically homogeneous entity. On the contrary, recent molecular pathogenomic studies have further revealed marked internal heterogeneity. Using mNGS to analyze 145 patients with spinal infection, Gao et al. found that NVO and iatrogenic vertebral osteomyelitis (iVO) differed not only in clinical background, but also in pathogen-spectrum composition. At the phylum level, Actinobacteria were more abundant in NVO, whereas Firmicutes were more enriched in iVO. At the genus level, iVO was mainly characterized by *Staphylococcus*, whereas *Mycobacterium* was more common in NVO (Gao et al., 2024). These findings suggest that future refinement of standardized NVO frameworks should integrate clinical, imaging, and microbiological evidence while also accounting for microbiological heterogeneity associated with different infection sources. This may further improve empirical treatment selection, biopsy strategies, and molecular testing pathways. Alongside this framework reconstruction, epidemiological surveillance has identified an increasing clinical concern regarding dual infection involving NVO and infective endocarditis (IE). Because these two conditions share overlapping causative pathogens, particularly *Staphylococcus aureus* and streptococci, concomitant infection is associated with considerable mortality risk. A long-term systematic follow-up study of patients with NVO complicated by IE reported an in-hospital mortality rate of 14.0% and a three-year mortality rate of 16.0% after diagnosis, which may still be underestimated because of loss to follow-up (Borgonovo et al., 2025). For these complex, hematogenously disseminated multifocal infections, early systemic evaluation, active screening for cardiovascular complications, and rigorous microbiological assessment through repeated blood cultures and targeted biopsy are particularly important. Increasing host complexity, microbiological variability, and predictors of treatment failure further highlight the need for early risk stratification, individualized management strategies, multidisciplinary evaluation, and close follow-up of high-risk patients to improve clinical outcomes (Matsuo et al., 2026).

### 8.2 Establishment of multidisciplinary care systems and the clinical value of the COMBINE interdisciplinary model

As the diagnosis of bone and joint infections enters the era of multimodal assessment, the traditional model in which diagnostic and therapeutic decisions depend primarily on the individual experience of orthopedic surgeons is increasingly insufficient for managing complex cases. This is particularly evident in the context of rapid advances in high-throughput molecular testing, antimicrobial resistance gene analysis, histopathological interpretation, and implant-associated biofilm research. How to effectively integrate heterogeneous evidence from different disciplines has become an important prerequisite for improving clinical outcomes. The COMBINE model from Denmark, reported in 2025, provides a representative example of interdisciplinary collaboration. Established in 2018, this center was designed to promote sustained collaboration between clinicians and basic researchers in the field of bone and joint infections. By integrating orthopedics, clinical microbiology, immunology, biofilm research, materials science, and related experimental disciplines, the COMBINE framework aims to facilitate bidirectional translation from pathogenetic understanding to intervention design (Jensen et al., 2025). The significance of such interdisciplinary platforms is not limited to research organization, but may also translate into tangible clinical benefits. A 2025 retrospective study involving 86 surgical cases of PJI showed that MDT participation was one of the strongest predictors of successful treatment among patients undergoing debridement, antibiotics, and implant retention (DAIR). The cure rate reached 100% in cases managed collaboratively by an MDT, compared with 48% in cases managed solely by the orthopedic team. The involvement of infectious disease specialists in antimicrobial optimization, refined interpretation of microbiological findings, and anti-biofilm treatment strategies may substantially improve the probability of DAIR success. Although these findings require further validation in larger prospective multicenter studies, they clearly suggest that the management outcome of complex bone and joint infections depends not only on the surgical approach itself, but also on the quality of perioperative pathogen assessment and antimicrobial strategy (Lucas et al., 2025). After the introduction of MDT management in specialized centers, patients with PJI may experience fewer repeat surgeries, shorter hospital stays, and improved DAIR outcomes (Vuorinen et al., 2021). Earlier studies on hip PJI showed that regular multidisciplinary infection meetings could significantly alter treatment plans originally made by a single specialty and thereby influence clinical outcomes (Ntalos et al., 2019). A qualitative study further indicated that PJI MDT meetings function by achieving interdisciplinary balance, integrating conflicting evidence, and sharing responsibility for high-risk decisions (Broom et al., 2023). In terms of team composition, a mature bone and joint infection MDT should generally include orthopedic surgeons, trauma surgeons, infectious disease specialists, and clinical microbiologists. In complex cases, the participation of radiologists, pathologists, plastic and reconstructive surgeons, pharmacists, and experimental researchers may also be important. Relevant studies have shown that orthopedic surgeons, infectious disease physicians, and clinical microbiologists have the highest attendance rates in complex BJI MDT meetings (Hanssen et al., 2025). This suggests that the value of MDTs does not lie simply in increasing the number of participants, but in enabling early collaboration among infectious disease, microbiology, pathology, and surgical teams to improve case stratification, sample interpretation, and individualized antimicrobial therapy.Therefore, an effective MDT should function as a dynamic collaborative network built around sample interpretation, antimicrobial optimization, surgical strategy, and long-term follow-up. The interdisciplinary framework represented by COMBINE, together with the gradual maturation of MDT-based PJI management, is jointly driving the diagnosis and treatment of bone and joint infections away from specialty-isolated decision-making and toward integrated, multi-evidence-based management.

## 9 Clinical limitations and future directions from a global health perspective

### 9.1 Technological gaps and unequal resource allocation in low- and middle-income countries

Although theoretical advances and high-end omics technologies have achieved unprecedented progress in leading medical centers in Europe and North America, the real-world implementation of advanced laboratory diagnostics across routine global clinical practice still faces substantial economic and standardization barriers (Tissingh et al., 2022; Fonkoué et al., 2026). From a global health perspective, low- and middle-income countries (LMICs) often bear a higher burden of open injuries, delayed presentation, and infection-related complications, while having the weakest laboratory diagnostic capacity. A prospective multicenter study from Cameroon involving 169 patients with fracture-related infection (FRI) across four tertiary hospitals in Yaoundé showed a clear gap between current practice and international consensus recommendations. Notably, 34.3% of cases had no previous surgical history, which also limits the direct applicability of some guidelines developed primarily around implant-associated infections in these settings (Fonkoué et al., 2026). A review of the current status of orthopedic infection laboratory diagnosis in Nepal similarly noted that, outside major cities, laboratory support remains limited in many regions, and empirical treatment, delayed etiological identification, and non-standardized antimicrobial use are still common (Bhatta et al., 2021; Khadka et al., 2025). This technological gap does not merely refer to the absence of mNGS or multiplex PCR, but also reflects the fragility of the most fundamental diagnostic chain. International consensus recommendations suggest that 3–5 independent deep tissue samples should be collected intraoperatively for standard culture in suspected FRI (Dudareva et al., 2019). When feasible, pathogen detection can be further improved by combining enrichment culture, inoculation into blood culture bottles, sonication, or molecular testing (Bellova et al., 2021). However, in many resource-limited settings, the real challenge is whether samples can be collected properly, transported in time, and processed by laboratories with adequate capacity. Conventional culture remains the foundation of etiological diagnosis in FRI, whereas molecular testing, although promising, is still clearly constrained by cost in routine application (Lourtet-Hascoët et al., 2025). Another challenge in LMICs is that pathogen spectra and antimicrobial resistance patterns may differ from those in high-income countries. In low-resource settings, the distribution of causative organisms is not identical to that in high-resource regions, with a higher proportion of Gram-negative bacteria and a substantial antimicrobial resistance burden. In some low-resource countries, the proportion of multidrug-resistant Gram-negative organisms in FRI may be significantly higher than that reported in high-income settings (Tissingh et al., 2022; Nokwe et al., 2026). Therefore, the future priority for LMICs should not be framed as simply catching up with every frontier technology. Instead, greater emphasis should be placed on resource-stratified diagnostic upgrading. Tools that are low-cost, rapid, and less dependent on complex laboratory infrastructure may have greater practical relevance. For example, in PJI, the synovial calprotectin lateral flow test (LFT) has relatively high diagnostic accuracy and better accessibility, making it particularly suitable as a rapid rule-out tool (Festa et al., 2024). Similarly, point-of-care tests such as alpha-defensin, leukocyte esterase, and calprotectin may provide more practical incremental value in settings where complex molecular platforms are not routinely available (Altsitzioglou et al., 2024). In regions with a high tuberculosis burden, GeneXpert has shown high sensitivity and moderate specificity in the diagnosis of spinal tuberculosis and may serve as a useful adjunct to conventional culture and routine histopathology (Biakto et al., 2024). Truly globally accessible innovation is not necessarily represented by the most expensive technology, but by diagnostic strategies that optimize the balance between clinical gain and cost in resource-limited environments.

From an implementation perspective, more feasible strategies may include the following. First, a basic resource-stratified algorithm should be established, prioritizing standardized deep-sample collection, withholding antibiotics before sampling when clinically appropriate, basic culture, and histopathological interpretation. Second, reproducible intermediate-level technologies, such as blood culture bottle inoculation, enrichment culture, simplified molecular assays, and tuberculosis-related nucleic acid platforms, should be gradually deployed in regional centers. Third, for a limited number of highly complex or culture-negative cases, regional referral or centralized testing networks should be established, rather than requiring every hospital to independently maintain a full high-throughput diagnostic platform. Existing reviews and practical studies from low-resource settings suggest that diagnostic performance is determined not only by the equipment itself, but also by whether standardized workflows, sample quality, transport conditions, and antimicrobial stewardship are optimized simultaneously. From a broader frontier perspective, the next step in global health is not simply to transfer algorithms developed in high-income countries to LMICs, but to develop diagnostic systems that are scalable, maintainable, and locally validated. This may involve developing more affordable synovial fluid POCT tools, calibrating local biomarker cut-off values, designing targeted molecular panels around highly prevalent pathogens, and using implementation science to evaluate which diagnostic strategies can truly improve rational antimicrobial use, shorten diagnostic delays, and reduce treatment failure in resource-limited environments. Only when diagnostic innovation moves beyond technical feasibility toward global accessibility can the upgrading of laboratory diagnosis for bone and joint infections achieve genuine public health significance.

### 9.2 Toward *In Situ* monitoring and non-invasive precision assessment

Traditional diagnosis of bone and joint infections has long been constrained by a pathway dependent on invasive sampling, ex vivo testing, and delayed feedback. Therefore, future breakthroughs are unlikely to arise solely from the addition of individual new biomarkers, but may instead depend on a transformation in the mode of monitoring itself. In other words, diagnostic strategies are expected to move gradually from static, ex vivo, and terminal time-point testing toward *in situ*, dynamic, and minimally invasive assessment. One of the most representative current directions is real-time microenvironmental sensing at the implant interface. Recent studies have integrated pH-sensitive films directly onto implant surfaces and combined them with X-ray-excited luminescence chemical imaging (XELCI) to achieve non-invasive imaging through soft tissue. In preclinical rabbit models, this technology has been able to capture acidification signals on implant surfaces, suggesting its potential to identify local microenvironmental abnormalities caused by bacterial colonization and biofilm formation before overt clinical symptoms or typical imaging changes become apparent (Uzair et al., 2025). Although this strategy remains some distance from routine clinical application, it represents an important shift in BJI diagnosis from detecting late-stage consequences to monitoring early interface-level events. Completely non-invasive body-fluid monitoring represents another future direction of interest. Among these approaches, urinary peptide biomarkers are particularly conceptually meaningful. The theoretical basis is that protein degradation fragments generated by infection-related tissue damage and host inflammatory responses can be filtered by the kidneys and enter the urine, leaving analyzable molecular fingerprints in a body fluid distant from the infection site. Preliminary clinical studies have shown that classification models based on urinary peptide profiles may have favorable diagnostic performance in distinguishing PJI from aseptic loosening. However, current evidence remains limited to small-sample, single-center studies and lacks cross-platform reproducibility assessment and large-scale external validation (Omar et al., 2021). Therefore, this approach is more appropriately viewed as a future non-invasive adjunct rather than a near-term replacement for local sample-based testing. Another emerging area in non-invasive diagnosis may come from infection-specific molecular imaging rather than further refinement of traditional anatomical imaging. Recent studies have begun to explore PET tracers with greater pathogen or host-response specificity in order to overcome the limited discriminatory ability of conventional ^18F-FDG in differentiating sterile inflammation from infection (Pollard-Kerning et al., 2024; Mdanda et al., 2026). Although these technologies remain largely at the preclinical or early translational stage, their potential value lies in the possibility of not only determining the presence of infection, but also providing information on infection activity, spatial distribution, and dynamic treatment response. In this sense, diagnosis, therapeutic assessment, and follow-up monitoring may gradually become more integrated.

Not all emerging technologies, however, belong to the category of non-invasive future diagnostics. Dithiothreitol (DTT), for example, mainly acts at the stage of ex vivo sample processing. By disrupting biofilms and the viscous synovial matrix, DTT can improve pathogen release and recovery from synovial fluid or implant-associated samples, thereby enhancing the detection performance of subsequent culture or molecular assays (Sambri et al., 2018; Tsikopoulos et al., 2022). ICM 2025 has recognized DTT as a biofilm-disruption strategy with practical potential, but it should be classified as an optimization method for sample pretreatment rather than an *in situ* monitoring technology. In other words, the future evolution of BJI diagnosis will not follow a single pathway, but will likely involve a multilayered upgrade composed of *in situ* sensing, non-invasive biomarkers, and ex vivo sample-enhancement technologies (Jennings et al., 2026). Overall, the true transformation of future BJI diagnostics will not merely involve making tests more sensitive, but rather making them earlier, less invasive, more dynamic, and more closely aligned with the *in situ* processes occurring at the implant–host–pathogen interface. Within this framework, implant-interface chemical sensing, non-invasive monitoring approaches such as urinary peptide profiling, and infection-specific molecular imaging represent different exploratory directions across local, systemic, and whole-body spatial scales. Whether these technologies can ultimately enter routine clinical practice will depend on standardization, external validation, cost control, and their ability to integrate with existing diagnostic algorithms. (**Table 6**).

**Table 6:** Frontier directions, translational potential, and practical barriers in the laboratory diagnosis of bone and joint infections.

Frontier direction	Core content	Potential clinical value	Current major barriers
Host immune proteomics	Mapping local immune protein profiles using LC-MS/MS, Olink, and related platforms	Differentiates PJI from non-infectious arthroplasty failure (NIAF), identifies host inflammatory signatures, and expands prognostic stratification	High cost, strong platform dependence, and lack of standardized thresholds
Host-specific antibody targets	Antibody responses against targets such as IsdB and Gmd	Extends laboratory testing from determining “whether infection is present” to predicting outcome risk	Insufficient target specificity, limited external validation, and challenges in clinical implementation
Host transcriptomics/multiplex mRNA platforms	Detection of early host immune signals and bacterial transcripts	Provides highly sensitive detection potential for culture-negative and early-stage infections	Standardization challenges, high cost, and complex interpretation
Microbiome/gut–bone immune axis	Association between gut microbiome dysbiosis and susceptibility to implant-associated infection	May support risk prediction and perioperative intervention	Mechanisms remain incompletely defined and clinical evidence is still insufficient
Integrated AI/ML models	Integration of laboratory, imaging, omics, and clinical variables	Improves risk stratification and diagnostic consistency in complex cases	Mostly based on single-center retrospective studies, insufficient external validation, and limited interpretability
IMC + deep learning	Identification of infection and pathogen type based on heat-flow curves	Enables rapid qualitative diagnosis within 24 hours	Limited equipment availability and still an emerging technology
NVO standardization framework	Unification of NVO definitions and diagnostic pathways	Reduces heterogeneity and improves comparability across studies and centers	Pathogen spectra still differ substantially across infection sources
MDT/COMBINE model	Collaboration among orthopedics, infectious diseases, microbiology, pathology, and related disciplines	Improves DAIR success, sample interpretation, and treatment decision-making	Dependent on centralized resources and structured workflow development
Resource-stratified diagnostics in LMICs	POCT, simplified molecular testing, and regional centralized testing networks	Improves global accessibility and public health impact	Unequal resources, non-standardized sampling workflows, and cost constraints
*In situ* monitoring and non-invasive diagnosis	XELCI, urinary peptide profiling, infection-specific PET, and related approaches	Shifts diagnosis from static ex vivo testing toward dynamic and minimally invasive monitoring	Mostly at preclinical or early translational stages, with insufficient external validation

## 10 Conclusion

The laboratory diagnosis of bone and joint infections has evolved from reliance on isolated serum markers or conventional culture toward disease-specific, specimen-based, and composite diagnostic interpretation. Serum biomarkers remain useful for initial screening and dynamic monitoring, but they are insufficient for standalone diagnostic confirmation. Synovial fluid, deep-tissue, bone biopsy, and sonication-based testing can more directly reflect the infection-site microenvironment and etiological status when interpreted within the appropriate disease context. Multiple deep-sample collection, histopathological assessment, and sonication remain important diagnostic foundations, whereas targeted multiplex PCR, broad-range 16S rRNA gene PCR/qPCR, targeted sequencing, and untargeted shotgun mNGS provide adjunctive opportunities for selected culture-negative, low-virulence, antibiotic-exposed, or complex polymicrobial infections. The field has already moved beyond the search for a single definitive biomarker, particularly in PJI, where MSIS/ICM and EBJIS frameworks integrate serum markers, synovial fluid parameters, microbiology, histopathology, and intraoperative findings, and in FRI, where consensus definitions distinguish confirmatory from suggestive criteria. Building on this foundation, future progress in BJI diagnosis should focus on refining and externally validating disease-specific diagnostic algorithms, standardizing sampling procedures, improving local and molecular adjuncts, and integrating emerging AI- or ML-assisted tools with existing composite criteria and MDT-based interpretation. Such a strategy may enable earlier, more accurate, and more clinically actionable diagnostic decision-making across heterogeneous BJI entities.
